# Antioxidant HPTLC-DPPH Fingerprinting of Honeys and Tracking of Antioxidant Constituents upon Thermal Exposure

**DOI:** 10.3390/foods10020357

**Published:** 2021-02-07

**Authors:** Md Khairul Islam, Tomislav Sostaric, Lee Yong Lim, Katherine Hammer, Cornelia Locher

**Affiliations:** 1 Cooperative Research Centre for Honey Bee Products Limited (CRC HBP), University of Western Australia, Myers Building, M087, Perth 6009, Australia; mdkhairul.islam@research.uwa.edu.au (M.K.I.); katherine.hammer@uwa.edu.au (K.H.); 2 Division of Pharmacy, School of Allied Health, University of Western Australia, Crawley 6009, Australia; tom@chromatechscientific.com (T.S.); lee.lim@uwa.edu.au (L.Y.L.); 3 School of Biomedical Sciences, University of Western Australia, Crawley 6009, Australia

**Keywords:** food analysis, antioxidant band activity, *Leptospermum*, *Eucalyptus*, Marri, degradation monitoring

## Abstract

The use of High-Performance Thin-Layer Chromatography (HPTLC) coupled with the use of DPPH* (2,2-diphenyl-1-picrylhydrazyl) as a derivatisation reagent is a novel approach to the analysis of antioxidant activity of honeys. The method facilitates the visualisation of individual constituents that contribute to the overall antioxidant activity of the honey, even if they are not yet chemically identified, and allows for the quantification of their antioxidant activity as gallic acid equivalents. The method supports a more in-depth study of the antioxidant activity of honey as it allows for a comparative analysis of the antioxidant fingerprints of honeys of different floral origin and is able to capture differences in their individual bioactive constituents. Further, it supports the tracking of changes in antioxidant activity of individual honey constituents over time upon exposure to different temperature conditions, which demonstrates the potential value of the method for in-process quality control.

## 1. Introduction

Honey, a highly concentrated complex mixture of mainly sugars (75–85%), water (13–21%), and a small fraction of non-sugar constituents (approx. 3%) [1,2,3], has been appreciated for centuries—not only as a food item, but also for its medicinal properties [4]. The earliest recorded medicinal use of honey dates back to the Ancient Egyptian era [5,6]. To date, a range of bioactivities have been investigated, amongst them antimicrobial, antioxidant, anti-inflammatory, anticancer, antidiabetic, anti-hyperlipidemic, anti-ulcer, as well as radical scavenging and wound-healing activities [7,8,9,10]. Next to its antibacterial effects, the antioxidant activity of honey is of growing interest—although, to date, only a few studies have investigated the specific constituents that are responsible for this effect [11]. 

Free radicals cause oxidative stress on living tissues by damaging biomolecules essential for cell vitality, and are thus implicated in many conditions, like inflammation, cardiovascular diseases, cancer, and neuro-degenerative diseases [12,13]. Antioxidants have the ability to counteract these damaging effects. Phytochemicals, such as phenolic acids and flavonoids, are believed to be strongly correlated to the antioxidant properties of natural products promoted for human health benefits [14,15]. Phenolic acids and flavonoids represent a significant share of the non-sugar constituents of honey, and as such, not only contribute to its colour and organoleptic characteristics, but also to its bioactivity, including its antioxidant properties [16]. Commonly, the honey’s colour is seen as an indication for activity—the darker the honey, the higher its total phenolics content, and by extension, also its antioxidant properties [17,18]. Depending on the honey’s floral source, its phenolic acid and flavonoid profile can vary significantly. Thus, in particular for monofloral honeys, their phenolics profile is a distinguishing feature that will impact the honeys’ organoleptic characteristics and bioactivity levels, and with this, ultimately also their quality and the price they can yield [19].

While in general, the presence of phenolic constituents has been correlated with a honey’s antioxidant activity, detailed studies on the chemical nature of these phenolics and investigations on the potential contribution of non-phenolics to the overall antioxidant activity are sparse. Further, only limited comparative analyses between honeys have been carried out with respect to their antioxidant effects [11]. Depending on a honey’s constituent profile, different levels of antioxidant activity can be anticipated. Further, it can also be assumed that honeys, which are comparable in their overall antioxidant activity, might not necessarily have identical bioactive constituent profiles. It is therefore beneficial to visualise and quantify the various antioxidant phytochemicals that are present in honeys, even if their chemical identity is not yet known, and to establish similarities and differences in the respective activity profiles of different monofloral honeys.

Maintaining the level of antioxidant constituents present in honeys throughout the production, transport, and storage chain is of concern to the beekeeping industry, as it will ensure the ongoing quality of its products. Antioxidant assays can support a better understanding of the way these processes might affect bioactivity. Among the various antioxidant assays that exist, the DPPH assay is one of the most popular spectrophotometric assays for the determination of the total antioxidant activity of honeys [20,21]. DPPH* (2,2-diphenyl-1-picrylhydrazyl) is a stable free radical, which is sensitive to reaction with Lewis bases. It is characterised by an intense purple colour (absorbance at 515–520 nm), which is lost upon reaction with oxidising reagents. In particular, constituents which can rapidly decrease the absorbance of DPPH* by donating a hydrogen atom are considered good antioxidants. However, a significant advantage of the assay is that DPPH* reacts even with weak antioxidants if sufficient reaction time is given [22,23].

High-Performance Thin-Layer Chromatography (HPTLC) is a simple but increasingly popular tool, used for the analysis of natural products like honey [24,25,26]. It allows for the separation, and potentially also the quantification of various extract constituents using a semi-automated set-up. In this study, the method is coupled with DPPH* derivatisation [27] and applied to a range of commercially available honeys from Western Australia (Table 1) to determine commonalities and differences in their individual antioxidant constituent profiles. Further, it is demonstrated how HPTLC-DPPH fingerprinting can be used to track honey constituents over time in order to determine the potential impact of processing stressors (e.g., elevated temperature) on the activity of the honey’s antioxidant constituents. This is relevant as honeys are, for instance, routinely warmed (between 55–80 °C) [28,29] to facilitate filtration to remove debris from raw honey and dispense into packaging jars. Furthermore, particularly in warmer climates, honeys might also be exposed to elevated temperatures during their storage. It has been demonstrated that heat exposure can impact on honeys’ antibacterial activity [30] and potentially also lead to the formation of unwanted by-products, such as hydroxymethylfurfural [31,32], but the effect on honeys’ antioxidant activity has not yet been studied to the same extent.

## 2. Materials and Methods

### 2.1. Chemicals and Reagents

Reagents were sourced from: 4,5,7-trihydroxyflavanone (Alfa Aesar, Lancashire, UK), gallic acid (Ajax Chemicals Ltd., Sydney, Australia), DPPH* (Fluka AG, Buchs SG, Switzerland), and anhydrous sodium sulfate (Merck KGaA, Darmstadt, Germany). Solvents were purchased from: Methanol (Scharlau, Barcelona, Spain), dichloromethane (Merck KGaA, Darmstadt, Germany), toluene (APS Chemicals, Sydney, Australia), vanillin (Sigma-Aldrich, St. Louis, MO, USA), ethyl acetate, and formic acid (Ajax Finechem Pvt Ltd., Sydney, Australia). Commercial honeys (Table 1) were obtained from supermarkets and honey suppliers in Western Australia. Where possible, the honey’s floral source was derived from the label, and no further authentication was carried out.

### 2.2. Sample Preparation

#### 2.2.1. Standard Solution and Reagent Preparations

A standard stock solution of gallic acid (20 µg/mL) in methanol and a reference solution of 0.5 mg/mL of 4,5,7-trihydroxyflavanone in methanol were prepared. A mixture of toluene: ethyl acetate: formic acid (6:5:1, *v*/*v*/*v*) was used as the mobile phase [33]. The derivatisation reagent was prepared by dissolving 40 mg DPPH* in 10 mL of 50% methanol and 50% ethanol and stored in an amber glass bottle, protected from light, until use [34]. A vanillin spraying reagent was prepared by adding 2 mL of sulfuric acid to 100 mL vanillin solution (1 g/100 mL in ethanol).

#### 2.2.2. Honey Sample Preparation

For the analysis, 1 g of each commercial honey was mixed with 2 mL of deionised water in a glass stoppered tube, and then vortexed to produce a homogenous solution. The aqueous honey solutions were then extracted three times with 5 mL of dichloromethane. The combined organic extracts were dried with anhydrous MgSO_4_, filtered, and evaporated to dryness at room temperature. The dried extracts were stored at 4 °C until further analysis. Prior to HPTLC analysis, the samples were reconstituted in 100 μL of dichloromethane.

### 2.3. Chromatography

Two plates were prepared for the analysis, where one was used to visualise and quantify antioxidant honey constituents, and the other to obtain the honey’s floral fingerprint [35]. 

#### 2.3.1. Sample Application

For the quantification of antioxidant honey constituents as gallic acid equivalents, 4 µL of the reference solution, 4 µL of the gallic acid standard solution, and 5 µL each of the respective honey extracts were applied as 8 mm bands at 8 mm from the lower edge of the HPTLC plate at a rate of 150 nL s^−1^ using a semi-automated HPTLC application device (Linomat 5, CAMAG). To prepare a gallic acid standard curve in the honey matrix, 2 µL, 3 µL, 4 µL, 5 µL, 6 µL, and 7 µL of gallic acid standard solution were applied by over-spotting the respective honey bands. To obtain the honeys’ floral fingerprint, only honey extracts (5 µL each) were applied to the plate.

#### 2.3.2. Development

For each plate, the chromatographic separation was performed on silica gel 60 F_254_ HPTLC plates (glass plates 20 cm × 10 cm, Merck, Darmstadt, Germany) in a saturated (33% relative humidity), automated development chamber (ADC2, CAMAG). The plates were pre-saturated with the mobile phase for 5 min, automatically developed to a distance of 70 mm at room temperature, and dried for 5 min. The obtained chromatographic results were documented using a HPTLC imaging device (TLC Visualizer 2, CAMAG) under white light. The chromatographic images were digitally processed and analysed using specialised HPTLC software (visionCATS, CAMAG), which was also used to control the individual instrumentation modules [25,33,35].

#### 2.3.3. Derivatisations

After initial documentation of the chromatographic results, the first plate was derivatised with 3 mL of 0.4% DPPH* reagent (CAMAG Derivatizer). The derivatised plates were again analysed with the HPTLC imaging device under white light, and images were taken 60 min after derivatisation.

To obtain the floral fingerprints, the second plate was derivatised with 3 mL of vanillin spraying reagent (CAMAG Derivatizer). The derivatised plate was heated (CAMAG TLC Plate Heater III) at 100 °C for 3 min until colour developed before being analysed (TLC Visualizer, CAMAG) under white light and 366 nm.

#### 2.3.4. Data Analysis and Statistics

For the quantification of antioxidant honey constituents as gallic acid equivalents, the obtained images were converted into individual absorbance points according to their Rf values. Using Excel©, the obtained data were converted into chromatograms, which were used to derive calibration curves of area of absorbance vs. concentration [27]. All experiments were performed in triplicates, and the results were evaluated by a one-way analysis of variance (ANOVA) followed by Tukey’s honestly significant difference (TukeyHSD) test, where a *p*-value of less than 0.05 was considered statistically significant. All statistical analyses were performed using Microsoft Office 365, R and R studio [36].

### 2.4. Validation

The quantification of antioxidant activity of honey extracts using HPTLC in combination with DPPH* derivatisation is fully validated, as described previously [27]. In brief, data were generated by plotting the peak area vs. applied amount of gallic acid standards in a range of 40–140 ng/band. Linearity of the assay was calculated based on the regression equation and correlation (r^2^) coefficient. The sensitivity, expressed as the limit of detection (LOD) and limit of quantification (LOQ), was based on the comparison of the standard deviation (SD) and the slope of the calibration curve, and was found to be 16.5 ng and 50 ng, respectively. The accuracy of mean recoveries was found to be in the range of 99.89–101.45%. Precision as intra-day precision was determined by analysing replicates of three known concentrations in triplicate (*n* = 3) within a single day. Precision as inter-day precision was determined by repeating the same experiment on different days. Variance between replicates was expressed as relative standard deviation (%RSD), and the obtained values (1.01–2.52% RSD) were within the acceptance range.

### 2.5. Thermal Exposure

Honey samples were placed in glass jars and kept at a constant temperature of 40 °C and 60 °C, respectively, using a temperature-controlled heating oven (Memmert GmbH + Co. KG, Büchenbach, Germany). Sampling was carried out at 0 min, 12 h, 24 h, and 48 h. The honey samples were extracted, their floral fingerprints and antioxidant profiles recorded, and their antioxidant band activities calculated according to the process described earlier.

## 3. Results and Discussion

### 3.1. Antioxidant Band Activity of Honeys

**Figure 1:**
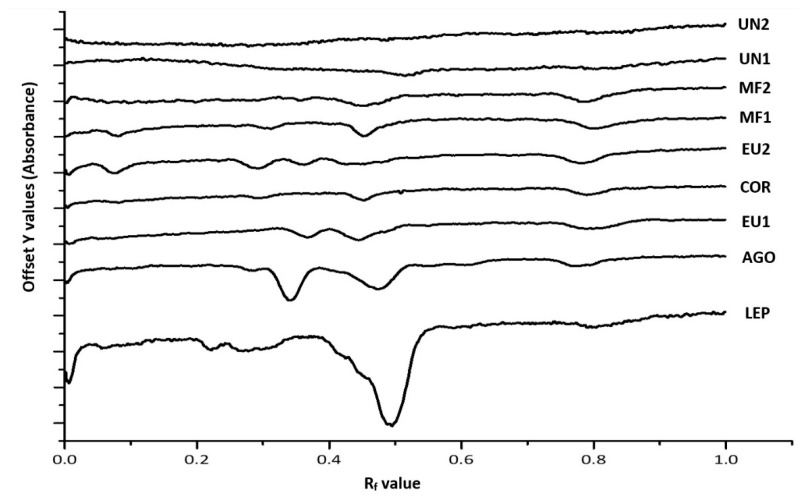
Organic honey extract chromatograms; 5 µL of each honey extract.

The chromatograms of the HPTLC images obtained for the various organic honey extracts after derivatisation with DPPH* reagent is presented in Figure 1. The antioxidant activity of bands was quantified and expressed as mg gallic acid equivalents per 100 g of honey (Table 2).

The trends found for antioxidant activity of the investigated honeys were similar to other studies, where the total antioxidant activity using DPPH* reagent was reported [37,38]. For example, the honey labelled as Manuka honey (LEP) displayed the highest level of activity in this study, and it was also reported as a highly antioxidant honey by others [39]. 

The value of this method is that it provides a more nuanced picture by capturing the antioxidant activity of individual honey constituents. For example, the honey labelled as Marri (COR) and the unspecified organic honey extract (MF1) displayed very similar total band antioxidant activity, but the HPTLC–DPPH analysis could demonstrate that only two bands (Rf 0.42 and 0.81) were mainly responsible for the antioxidant activity of the Marri honey extract (COR), whereas four different constituents (at Rf 0.09, 0.31, 0.48, and 0.81) contributed to the total band activity of the organic honey extract (MF1) (Table 2). Thus, the findings demonstrate that honeys differ in their antioxidant profiles, despite displaying similar total band activity in the HPTLC–DPPH assay.

It also appears that there might be common antioxidant constituents present across different honey organic extracts illustrated, for example, by common bands at Rf 0.806–0.809 for COR and MF2, whereas the presence of other antioxidant constituents seems to be more tied to particular honeys. This can be seen, for instance, for the band at Rf 0.476 in LEP, which notably contributes to the extract’s antioxidant activity, but does not seem to occur across most of the other honeys analysed in this study. 

All monofloral honey extracts, except the one derived from Karri honey (EU1), displayed relatively high levels of antioxidant activity, which might be a reflection of their various phenolic and flavonoid constituents. However, it is interesting to note that not all honeys from unspecified floral sources were of the same antioxidant quality. Some of them displayed high levels (i.e., MF1 and MF2), whereas others (i.e., UN1 and UN2) had very little activity. A likely explanation is that the former two were produced by local beekeepers by mixing honeys of unspecified floral origins that might have carried some antioxidant activity. Honeys without a specified nectar source (UN1, UN2), on the other hand, only displayed negligible levels of antioxidant activity. 

### 3.2. Comparison of Honey Extracts’ Floral Fingerprints with Antioxidant Fingerprints

**Figure 2:**
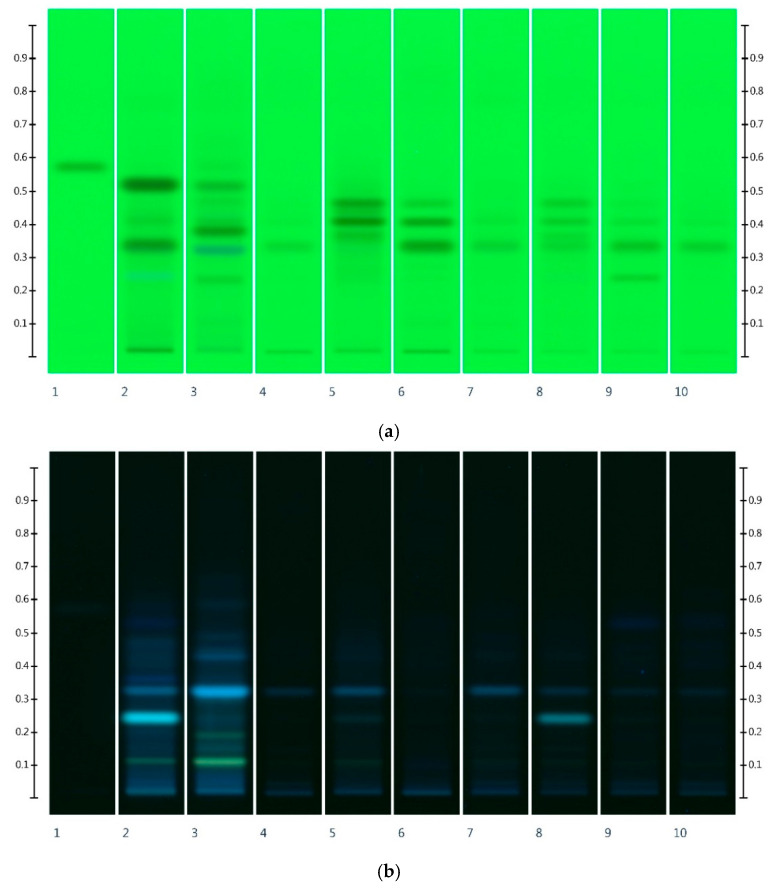

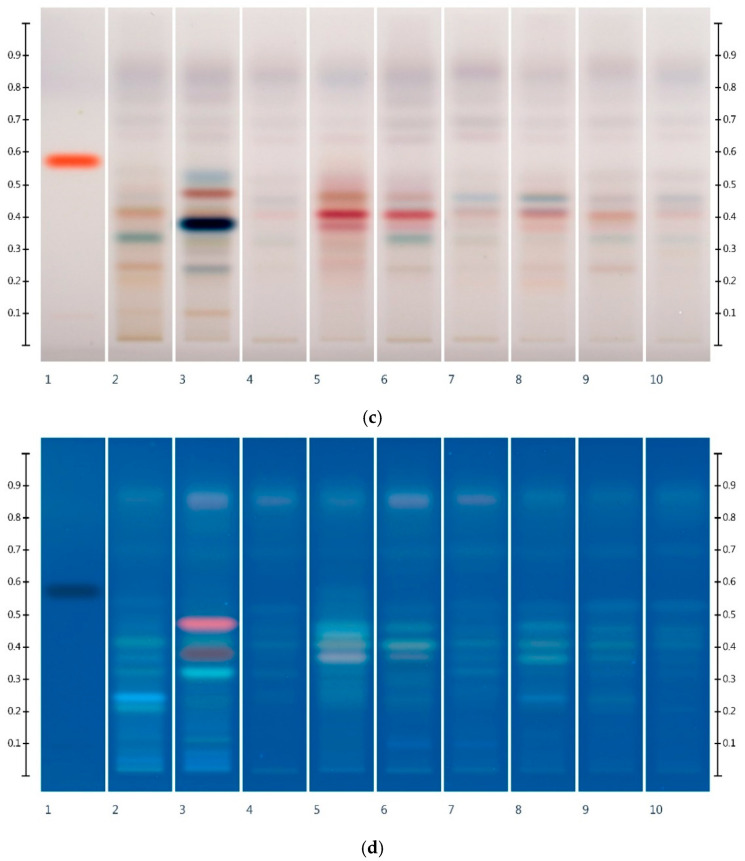
Honey extracts’ floral fingerprints. (**a**) Images taken at R 254 after development, (**b**) R 366 after development, (**c**) white light after derivatisation with vanillin reagent, and (**d**) R 366 after derivatisation with vanillin reagent; Track 1—4,5,7-trihydroxyflavanon, Track 2—LEP, Track 3—AGO, Track 4—EU1, Track 5—COR, and Track 6—EU2, Track 7—MF1, Track 8—MF2, Track 9—UN1, and Track 10—UN2; 5 µL of each honey extract.

It is also interesting to compare the honey extracts’ floral fingerprints, which are characteristic for their respective nectar sources (Figure 2), with the obtained antioxidant fingerprints (Figure 1). The HPTLC method, for the authentication of a honey’s floral source based on floral fingerprints, was published earlier [33,35]. A direct comparison between the respective fingerprints demonstrates that constituents signifying a honey’s floral source for authentication purposes might not necessarily contribute to its antioxidant activity. Table 3 directly compares the most important bands for authentication of the floral source with those considered key to the honey extract’s antioxidant activity.

### 3.3. Tracking of Thermal Stability of Selected Honeys

**Figure 3:**
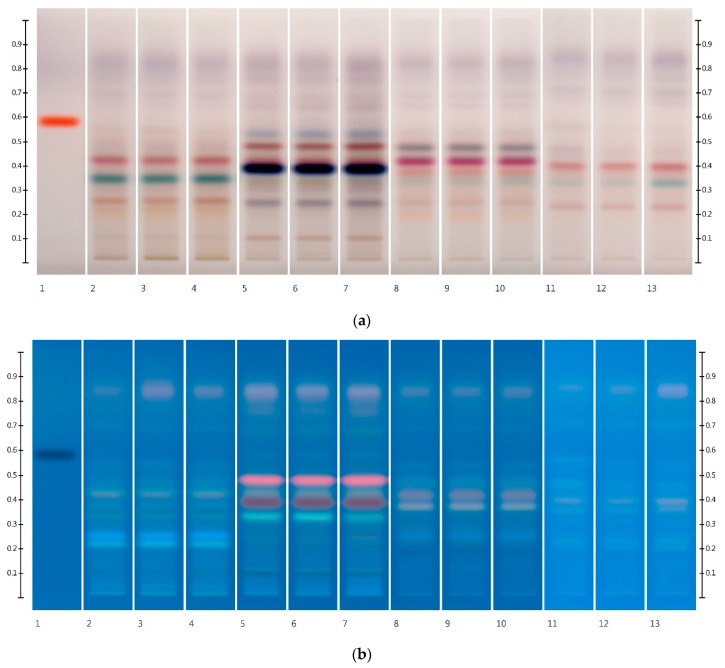
Floral fingerprints after thermal exposure: Images taken at (**a**) white light after derivatisation with vanillin reagent, and (**b**) R 366 after derivatisation with vanillin reagent; Track 1—4,5,7-trihydroxyflavanon, Track 2—LEP, Track 3—LEP at 40 °C after 48 h, Track 4—LEP at 60 °C after 48 h, Track 5—AGO, Track 6—AGO at 40 °C after 48 h, Track 7—AGO at 60 °C after 48 h, Track 8—MF2, Track 9—MF2 at 40 °C after 48 h, Track 10—MF2 at 60 °C after 48 h, Track 11—UN1, Track 12—UN1 at 40 °C after 48 h, and Track 13—UN1 at 60 °C after 48 h; 5 µL of each honey extract.

**Figure 4:**
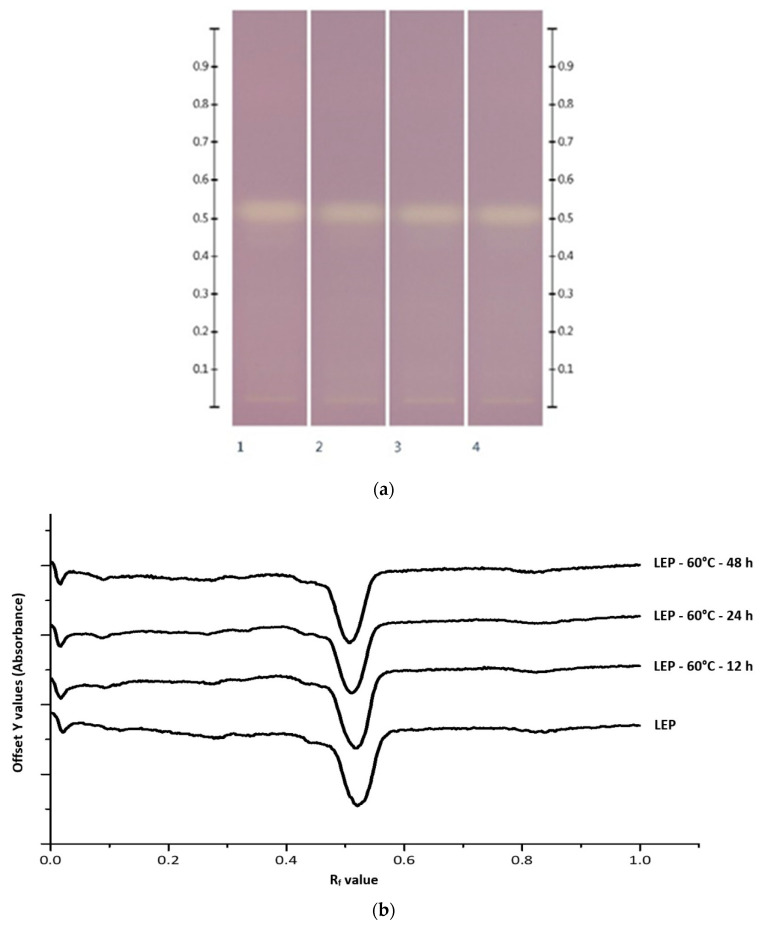
Antioxidant band activity after thermal exposure. Images taken at (**a**) white light after derivatisation with DPPH reagent and (**b**) their respective chromatograms; Track 1—LEP, Track 2—LEP at 60 °C after 12 h, Track 3—LEP at 60 °C after 24 h, Track 4—LEP at 60 °C after 48 h; 5 µL of each honey extract.

In order to explore the usefulness of HPTLC coupled with DPPH* derivatisation to track individual antioxidant honey constituents throughout their processing and storage, four different honeys, LEP, AGO, MF2, and UN1 (thus, two monofloral and two multifloral honey samples), were subjected to thermal stability testing, and their floral (Figure 3) and antioxidant (Figure 4, exemplary LEP only) fingerprints monitored for any potential changes. 

As can be seen from Figure 3, the investigated honeys appear to be relatively resistant to change in the chemical composition of their organic extracts on exposure to moderate temperatures (40–60 °C), as even a continuous exposure over 48 h had only minor effects on the honeys’ HTLPC profiles. 

HPTLC analysis coupled with DPPH* derivatisation allowed us to quantify the potential effect of heating on antioxidant band activity. As can be seen in Figure 4 exemplary for Manuka honey, some of the band intensities changed over 48 h of thermal exposure, which ultimately also impacted the honey’s total band activity. 

Thermal exposure experiments were conducted in triplicate for each investigated honey (LEP, AGO, MF2, and UN1). For better comparison, antioxidant band activities at each sampling time point were expressed as percentage (%) antioxidant band activity of the value found at the start of the experiment. 

**Figure 5:**
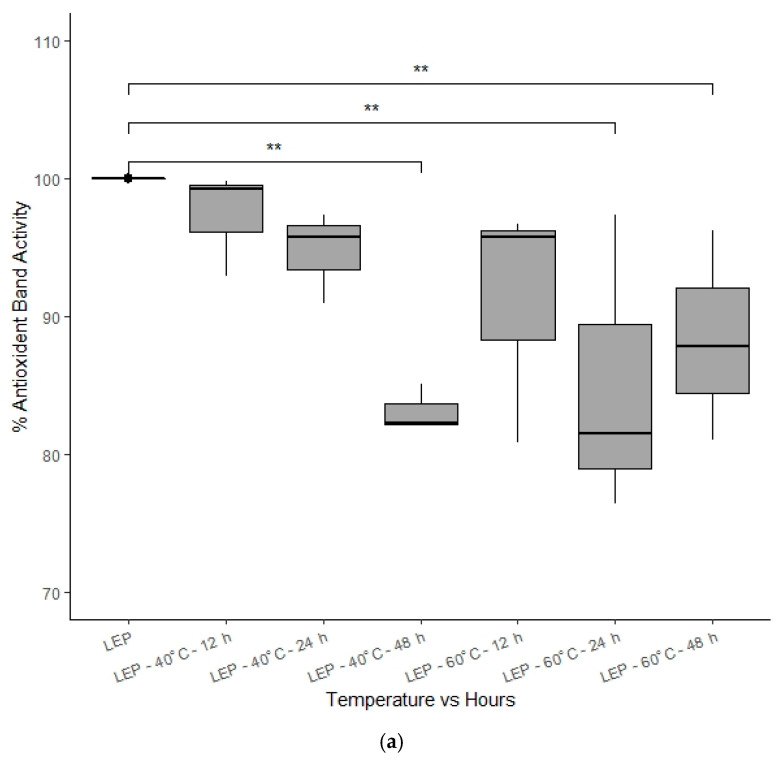

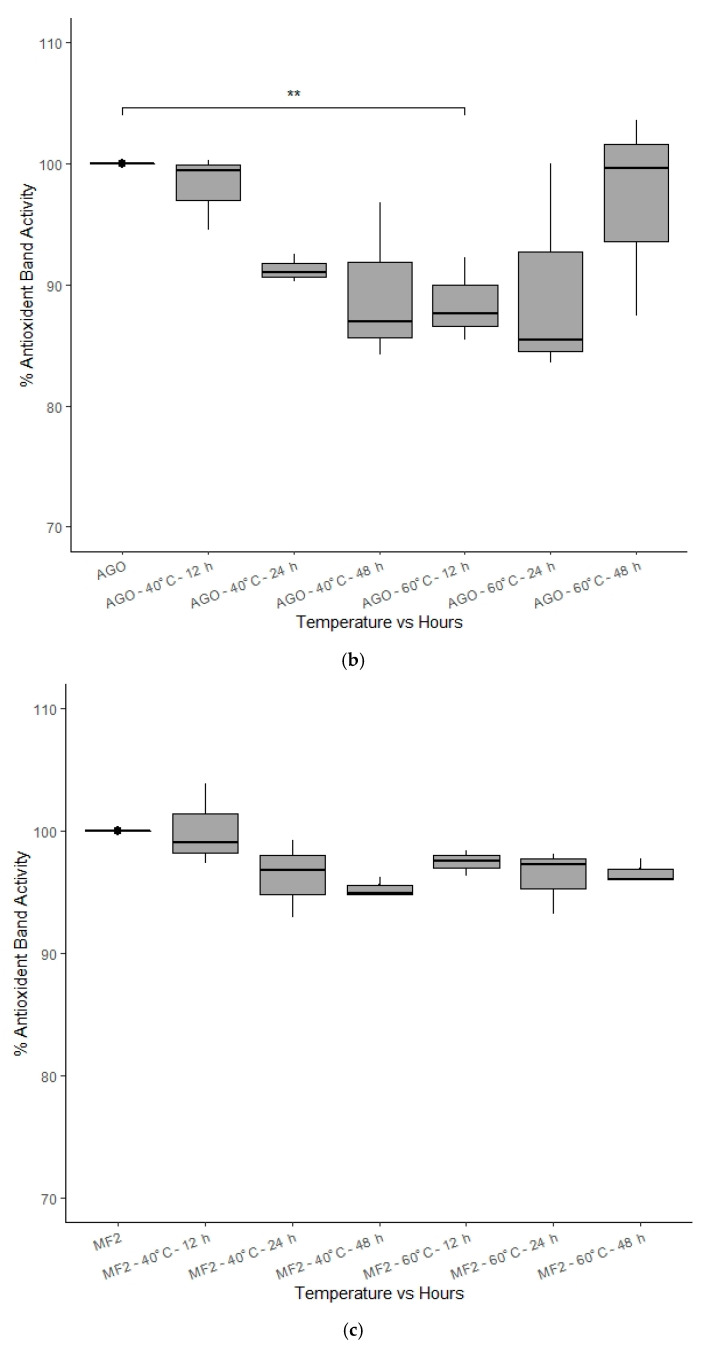

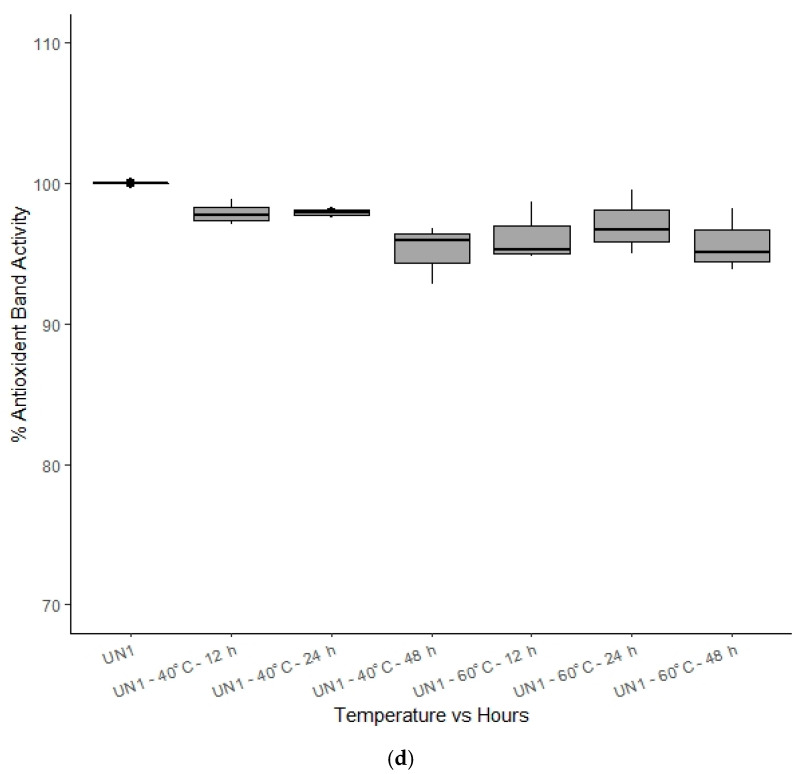
(**a**–**d**) Graphical representation of % antioxidant band activity of different honey extracts at different temperature time-points. Significance ** is indicated for *p* < 0.05.

Based on one-way ANOVA, statistically significant differences (*p* < 0.05) between various honeys exposed to different temperatures were found at different time points. Pair-wise ANOVA (TukeyHSD) revealed that MF2 and UN1 were stable in their % antioxidant band activity following exposure for up to 48 h at 40 °C and 60 °C (*p* values ranging between 0.994 and 1.000) (Figure 5c,d). In the case of AGO, no significant differences could be found at 40 °C (Figure 5b), whereas for LEP stored at 40 °C (Figure 5a), there were no changes in antioxidant band activity up to 24 h of heat exposure (*p* = 0.983), but at 48 h the honey’s antioxidant band activity was found to have significantly decreased to 83.16 ± 1.38% of the baseline value (*p* = 0.00004). Interestingly, % antioxidant band activity of AGO significantly decreased to 88.45 ± 2.83% of baseline (*p* = 0.0304) when stored at 60 °C for 12 h, but beyond 12 h, the % antioxidant band activity increased again (89.67 ± 7.33% at 24 h, *p* = 0.0996 and 96.88 ± 6.89% at 48 h, *p* = 0.999) (Figure 5b). A similar trend could be seen for LEP kept at 60 °C, where statistically significant differences were found beyond 12 h storage (decrease to 85.08 ± 8.91% at 24 h, *p* = 0.00056 and increase to 88.38 ± 6.20% at 48 h, *p* = 0.0282) (Figure 5a), which might be explained by the potential formation or release of antioxidant compounds over prolonged exposure to higher temperatures. The literature suggests, for example, that pollen, which is naturally present in honey, can degrade at higher temperature and release antioxidant compounds [18,40], which would then contribute to the overall antioxidant band activity. A more in-depth analysis of this phenomenon was, however, outside the scope of this study.

Overall, it appears that honeys with higher antioxidant band activities tend to be more affected by temperature, leading in most instances to a decrease of activity from baseline values, a finding which is also supported by other studies [29,40,41,42,43]. Additionally, as expected, a higher temperature seems to exert a stronger effect, as at 40 °C, most honeys were found to be stable, except LEP when exposed for 48 h. At the higher storage temperature of 60 °C, honeys with higher antioxidant band activities were affected even at shorter exposure times (beyond 12 h). More honeys would need to be examined to confirm the generalisability of these trends; however, this is outside the scope of this study, which does not aim to investigate particular honeys’ antioxidant characteristics, but to demonstrate the usefulness of HPTLC–DPPH fingerprinting for the tracking of antioxidant activity of honeys. 

## 4. Conclusions

High-Performance Thin Layer Chromatography (HPTLC) coupled with DPPH* derivatisation allows for a better understanding of the individual constituents responsible for a honey extract’s antioxidant activity. There are noticeable differences between honeys of different floral sources even if their total antioxidant band activity appears to be similar. Thus, the method offers both qualitative (antioxidant fingerprint) and quantitative (antioxidant band activity expressed as gallic acid equivalents) dimensions that help to differentiate between honeys of different botanical origin, and thus complement the honey extracts’ floral fingerprints. Next to more ubiquitous antioxidant constituents, unique phytochemicals also appear to be present in some of these honeys that contribute to their antioxidant properties. While their chemical identity has not yet been established, with the combination of HPTLC analysis and DPPH* derivatisation, their relative contribution to the extract’s antioxidant effect can nonetheless be quantified as gallic acid equivalents, and their potential change on exposure to processing stressors like heat can be documented. 

Given the increasing popularity of HPTLC analysis in natural product and food chemistry, it can be assumed that the combination of HPTLC with DPPH* derivatisation might also be a useful quality control tool for other natural products and food items where the maintenance of antioxidant activity throughout production and handling is also paramount.

## Figures and Tables

**Table 1:** Honey samples.

Labelled Floral Source	Nectar Source	Supplier and Packaging Date	Reference ID
Manuka	*Leptospermum* spp.	Barnes Naturals (January 2017)	LEP
Coastal Peppermint	*Agonis flexuosa*	Margaret River Honey Co. (December 2017)	AGO
Marri	*Corymbia calophylla*	ICON Honey (February 2018)	COR
Karri	*Eucalyptus diversicolor*	Zees Bees (2016)	EU1
River Red Gum	*Eucalyptus camaldulensis*	Capilano (May 2014)	EU2
Multifloral Organic	Unknown	Wescobee (December 2016)	MF1
Multifloral	Unknown	Wescobee (June 2019)	MF2
Unspecified	Unknown	Coles Supermarket (Jan 2019)	UN1
Unspecified	Unknown	Aldi Supermarket (No date provided)	UN2

**Table 2:** Antioxidant activity of individual bands in organic honey extracts.

ID	Bands	Rf	Concentration (ng/5 μL Extracts)	mg Gallic Acid Equivalent (per 100 g Honey)	Total Band Activity (mg Gallic Acid Equivalent per 100 g Honey)
LEP	1	0.27	80.32	0.1606	0.9685
	2	0.47	356.08	0.7122	
	3	0.83	47.85	0.0957	
AGO	1 *	0.35	352.04	0.7041	0.8101
	2 *	0.49			
	3	0.78	53.02	0.1060	
EU1	1	0.30	31.06	0.0621	0.2244
	2	0.46	34.46	0.0689	
	3	0.80	46.67	0.0933	
COR	1	0.43	113.67	0.2273	0.3615
	2	0.81	67.09	0.1342	
EU2	1	0.08	47.42	0.0948	0.5041
	2	0.41	129.55	0.2591	
	3	0.80	75.06	0.1501	
MF1	1	0.09	32.84	0.0657	0.3638
	2	0.31	34.08	0.0682	
	3	0.48	54.53	0.1091	
	4	0.81	60.47	0.1209	
MF2	1	0.46	64.50	0.1290	0.2527
	2	0.81	61.83	0.1237	
UN1	1	0.08	22.02	0.0440	0.1831
	2	0.51	51.13	0.1022	
	3	0.81	18.50	0.0369	
UN2	Not detected				

* Due to insufficient baseline separation, Bands 1 and 2 of AGO were quantitatively accounted for, capturing a Rf range of 0.264 to 0.670.

**Table 3:** Comparison between antioxidant bands and floral fingerprint bands of organic extracts of commercial Western Australian honeys.

ID	Antioxidant Bands (Rf)	Floral Fingerprint Bands (Rf)							
		After Development				After Derivatisation			
		R 254		R 366		T White		R 366	
		Rf	Colour	Rf	Colour	Rf	Colour	Rf	Colour
LEP	0.27	0.24	BRB	0.12	LY	0.25		0.20	BRB
	0.47	0.35		0.25	BRB	0.34	G	0.25	BRB
	0.83	0.52		0.33	LB	0.41	O	0.33	
						0.47	Y	0.42	
AGO	0.35	0.23		0.11	Y	0.10		0.11	
	0.49	0.33	BRB	0.19	LY	0.25		0.23	
	0.78	0.39		0.32	BRB	0.39	DB	0.32	G
		0.52		0.42	LB	0.47	BW	0.38	BW
								0.48	R
EU1	0.30	0.33		0.33	LB	0.32		0.32	
	0.46					0.41		0.40	
	0.80					0.46		0.52	
COR	0.43	0.37		0.25		0.37	P	0.25	
	0.81	0.41		0.32	LB	0.41	R	0.37	LBW
		0.47				0.47	O	0.41	DBW
								0.45	DB
EU2	0.08	0.34				0.24		0.10	B
	0.41	0.41				0.33	G	0.36	DBW
	0.80	0.46				0.40	R	0.40	DBW
						0.47	BW	0.46	DBW
MF1	0.09	0.34		0.33	LB			0.33	
	0.31					0.42		0.36	
	0.48					0.46		0.41	
	0.81								
MF2	0.46	0.33		0.11		0.20		0.23	B
	0.81	0.41		0.24	BRB	0.36	O	0.37	BW
		0.46		0.32	LB	0.41	LB	0.41	DBW
						0.46	B	0.47	DR
								0.52	
UN1	0.08	0.24				0.25	BW	0.37	
	0.51	0.34		0.23		0.33	G	0.41	BW
	0.81	0.40		0.32		0.40	BW	0.46	BW
								0.54	
UN2	Not Detected	0.33		0.31		Very faint; not easily distinguishable			

The Rf value of the standard (4,5,7-trihydroxyflavanon) was 0.58 (pink at T white light); Colour code: B—Blue, BW—Brown, R—Red, Y—Yellow, O—Orange, G—Green, P—Pink, LB—Light Blue, BRB—Bright Blue, DBW—Dull Brown, DB—Dark Blue, LY—Light Yellow.

## Data Availability

Not applicable.
